# Attachment Patterns of Human and Avian Influenza Viruses to Trachea and Colon of 26 Bird Species – Support for the Community Concept

**DOI:** 10.3389/fmicb.2019.00815

**Published:** 2019-04-18

**Authors:** Per Eriksson, Cecilia Lindskog, Victor Lorente-Leal, Jonas Waldenström, Daniel González-Acuna, Josef D. Järhult, Åke Lundkvist, Björn Olsen, Elsa Jourdain, Patrik Ellström

**Affiliations:** ^1^Zoonosis Science Center, Department of Medical Biochemistry and Microbiology, Uppsala University, Uppsala, Sweden; ^2^Department of Immunology, Genetics and Pathology, Science for Life Laboratory, Uppsala University, Uppsala, Sweden; ^3^Centre for Ecology and Evolution in Microbial Model Systems, Linnaeus University, Kalmar, Sweden; ^4^Facultad de Ciencias Veterinarias, Universidad de Concepción, Chillán, Chile; ^5^Zoonosis Science Center, Department of Medical Sciences, Uppsala University, Uppsala, Sweden; ^6^UMR0346 – EPIA, INRA, VetAgro Sup, Saint-Genès-Champanelle, France

**Keywords:** virus histochemistry, lectin staining, pattern of virus attachment, avian influenza, birds

## Abstract

Avian influenza A viruses (AIVs) have a broad host range, but are most intimately associated with waterfowl (Anseriformes) and, in the case of the H13 and H16 subtypes, gulls (Charadriiformes). Host associations are multifactorial, but a key factor is the ability of the virus to bind host cell receptors and thereby initiate infection. The current study aims at investigating the tissue attachment pattern of a panel of AIVs, comprising H3N2, H6N1, H12N5, and H16N3, to avian trachea and colon tissue samples obtained from host species of different orders. Virus attachment was not restricted to the bird species or order from which the virus was isolated. Instead, extensive virus attachment was observed to several distantly related avian species. In general, more virus attachment and receptor expression were observed in trachea than in colon samples. Additionally, a human seasonal H3N2 virus was studied. Unlike the studied AIVs, this virus mainly attached to tracheae from Charadriiformes and a very limited set of avian cola. In conclusion, the reported results highlight the importance of AIV attachment to trachea in many avian species. Finally, the importance of chickens and mallards in AIVs dynamics was illustrated by the abundant AIV attachment observed.

## Introduction

Influenza A viruses (IAVs) are pathogens of global concern in both human and veterinary medicine (Webster et al., 1992; Stöhr, 2002; Olsen et al., 2006; Wiethoelter et al., 2015). Wild birds are well-described hosts of avian influenza viruses (AIVs) and longitudinal surveillance studies have demonstrated a plethora of low pathogenic AIVs (LPAIVs) circulating in wild birds, particularly in dabbling ducks and other waterfowl (Krauss et al., 2004; Munster et al., 2007; Wallensten et al., 2007; Wille et al., 2011; Bahl et al., 2013; Huang et al., 2014; Arnal et al., 2015; Caron et al., 2017). Based on the wide host range it is suggested that IAVs are multi-host pathogens and there is clinical evidence of zoonotic transmission of either complete or reassorted AIVs to humans (Taubenberger et al., 1997; Fouchier et al., 2004; Garten et al., 2009; Watanabe et al., 2012; Qi et al., 2018). However, despite a large number of avian species screened in surveillance studies, there are only few species in which high AIV prevalence and subtype diversity is consistently detected (Krauss et al., 2004; Munster et al., 2007; Wallensten et al., 2007; Wille et al., 2011; Bahl et al., 2013; Huang et al., 2014; Arnal et al., 2015; Caron et al., 2017). This observation suggests that avian species differ in their capacity to maintain and transmit AIVs, and that certain species serve as reservoir species and others as spillover hosts with limited further transmission (Van Dijk et al., 2018). Surveillance studies alone cannot assess these questions but need to be complemented by experimental studies evaluating virus susceptibility and transmissibility.

Influenza A viruse subtypes H1-H16 are associated with aquatic birds, especially subtypes H1-H12 are associated with waterfowl (Wille et al., 2018). However, in contrast to subtypes H1-H7, which are frequently detected in waterfowl, subtypes H8-H12 are much less frequently reported. Among subtypes H8-H12, H9, and H10 stand out by having established maintained lineages in poultry. There is a massive turnover of LPAIVs in the wild bird population, illustrated by the seasonal pattern and annual subtype variability reported by surveillance studies (Krauss et al., 2004; Munster et al., 2007; Wallensten et al., 2007). However, despite the high theoretical number of possible hemagglutinin (HA) – neuraminidase (NA) combinations, only very few subtypes dominate the field isolate recordings including H3, H4, and H6. Moreover, subtypes H13 and H16 are mainly associated with gulls (Charadriiformes) indicating restrictions in AIV genetic exchange and host susceptibility (Wille et al., 2011; Huang et al., 2014). Additionally, subtypes H13 and H16 have recently been reported to be phenotypically distinguished from duck AIVs in terms of e.g., receptor and host cell tropism (Gambaryan et al., 2018).

Turnstones (*Arenaria*), belonging to the order Charadriiformes, are believed to play an important role in the ecology of AIVs in North America, but AIVs from this host have been characterized only to a limited extent (Krauss et al., 2010; Gambaryan et al., 2012). In particular, there is very little data available on AIVs of the H12 subtype (Wille et al., 2018). Moreover, H16 AIVs have mainly been isolated from members of Laridae and have only occasionally been reported from other avian families (Olsen et al., 2006; Wille et al., 2011; Gambaryan et al., 2018). How AIVs are maintained in wild avian hosts and the criteria of successful inter-species transmission remain key questions in AIV ecology. The prerequisites of this interplay are most probably multifactorial, including not only the host and the virus, but also the environment. A species barrier preventing AIVs to transmit to humans was postulated early (Scholtissek et al., 1985), including the distribution and linkage conformation of the IAV receptor molecule sialic acid (SA) at the host cell surface (Rogers and Paulson, 1983; Rogers and D’Souza, 1989; Ito et al., 1998). It is thus important to characterize the availability of AIV receptors and pattern of virus attachment (PVA) in different avian species to better understand the ecology of AIVs. In the present study, AIV attachment was investigated in a panel of bird tissue samples from both the New and Old Worlds, altogether comprising 26 different avian species, against an AIV panel of phylogenetically separated HA subtypes. To assess any potential differences in virus attachment between bird species, and to what extent such differences could be explained by host species origin of the viruses. Earlier studies have shown that AIVs are able to attach to human tissues (Van Riel et al., 2007; Lindskog et al., 2013; Eriksson et al., 2018), but less is known about AIV attachment in birds, the natural reservoir of IAVs, and only limited knowledge is available on IAV attachment in non-anseriform/non-charadriiform orders, as well as, any intra/inter order differences (Webster et al., 1992; Olsen et al., 2006; Caron et al., 2017). Trachea and colon tissues were investigated, since IAV infection is described as a gastrointestinal infection in ducks, whereas it causes respiratory infection in poultry, humans, and pigs (Olsen et al., 2006). Especially in mallards (*Anas plathyrynchos*), the most well-described wild bird LPAIV host species, AIV has been reported to be prominent in colon (Webster et al., 1978; Daoust et al., 2011; Costa et al., 2012; Bröjer et al., 2013; Lindskog et al., 2013). It was hypothesized that AIVs isolated from ducks would extensively attach to colon from waterfowl, whereas AIVs isolated from gulls would attach with reduced efficiency to non-charadriiform tissue, based on suggested attachment patterns reported by earlier studies (Webster et al., 1992; Olsen et al., 2006; Jourdain et al., 2011; Gambaryan et al., 2018).

## Materials and Methods

### Ethics Statement

Bird tissue sampling procedures were approved by the Swedish Environmental Protection Agency (permits numbers 412-6267- 08NV/412-5977-08NV), the Swedish Board of Agriculture (permit numbers 74-08/43-09), the Chilean Agriculture Ministry (permit number 1-25-2008), and the Ethics Committee of the Veterinary University of Concepción (permit number CE1-2006). All experimental procedures were performed in accordance with relevant guidelines and regulations.

### Tissue Preparation

To include avian tissue material from both the New and Old Worlds, trachea and colon samples were collected in Chile and Sweden from a set of bird species (Table 1). All sampled individuals were adult birds. The birds were not tested for AIV prior to sample collection. The avian tissue panel was designed to comprise both aquatic (Anseriformes, Suliformes, and Charadriiformes) and terrestrial orders (Galliformes, Columbiformes, Falconiformes, and Passeriformes) of birds. At least two individuals of each species were included, except for black-headed gull (*Larus ridibundus*), mew gull (*L. canus*), and elegant tern (*Thalasseus elegans*), for which only one individual was available for testing. Species with only a single representative were still included in the study, since an important aim of the study was to investigate whether any virus attachment could be observed in the studied avian species. Moreover, the tissue panel comprised tissues from rock pigeon (*Columba livia*) from both the New and Old Worlds (i.e., Chile and Sweden). Additionally, tissues from domestic chicken (*Gallus gallus*) were included to represent domestic poultry. Full details on the number of individuals per species and per tissue can be found in the Supplementary Table S1.

**Table 1:** Investigated avian species. Country of origin abbreviated according to ISO 3166-1.

Order	Family	Species	Common name	Sample origin
Galliformes	Phasianidae	*Gallus gallus*	Domestic chicken	SWE
Anseriformes	Anatidae	*Anser anser*	Greylag goose	SWE
		*Aythya fuligula*	Tufted duck	SWE
		*Mareca penelope*	Eurasian wigeon	SWE
		*Anas platyrhynchos*	Mallard	SWE
		*Anas georgica*	Yellow-billed pintail	CHL
Columbiformes	Columbidae	*Columba livia*	Rock dove	CHL/SWE
		*Zenaida auriculata*	Eared dove	CHL
		*Columbina picui*	Picui dove	CHL
Suliformes	Phalacrocoracidae	*Phalacrocorax brasilianus*	Neotropical cormorant	CHL
		*Phalacrocorax carbo*	Great cormorant	SWE
Charadriiformes	Charadriidae	*Vanellus chilensis*	Southern lapwing	CHL
	Laridae	*Larus ridibundus*	Black-headed gull	SWE
		*Larus pipixcan*	Franklin’s gull	CHL
		*Larus canus*	Mew gull	SWE
		*Larus dominicanus*	Kelp gull	CHL
		*Larus argentatus*	European herring gull	SWE
		*Thalasseus elegans*	Elegant tern	CHL
Falconiformes	Falconidae	*Phalcoboenus chimango*	Chimango caracara	CHL
Passeriformes	Corvidae	*Corvus corone*	Carrion crow	SWE
	Paridae	*Cyanistes caeruleus*	Eurasian blue tit	SWE
	Turdidae	*Turdus merula*	Eurasian blackbird	SWE
	Muscicapidae	*Erithacus rubecula*	European robin	SWE
	Regulidae	*Regulus regulus*	Goldcrest	SWE
	Passeridae	*Passer domesticus*	House sparrow	SWE
		*Passer montanus*	Eurasian tree sparrow	SWE

In brief, tissue specimens were formalin-fixed and paraffin-embedded, and used for generation of tissue microarrays (TMAs), as described previously (Kampf et al., 2012). Each individual sample was represented with duplicate 1 mm diameter tissue cores in the TMA. The TMA blocks were cut in 4 μm thick sections, mounted on adhesive slides (SuperFrost Plus, Thermo Fisher Scientific, Waltham, MA, United States) and baked for 45 min at 60°C prior to virus histochemistry staining.

### Virus Panel

The viruses in the AIV panel were isolated from mallard (*Anas platyrhynchos*) (A/Mallard/Sweden/68619/2007 [H3N2] and A/Mallard/Sweden/81/2002 [H6N1]), ruddy turnstone (*Arenaria interpres*) [A/Turnstone/Delaware/15/2007 (H12N5)], and black-headed gull (*Larus ridibundus*) [A/Black-headed gull/Sweden/2/1999 (H16N3)]. Mallards belong to Anseriformes and are the foremost-described host of AIVs, thus two AIVs of different subtypes isolated from mallards were included in the studied panel (Olsen et al., 2006). Moreover, two AIVs isolated from Charadriiformes were included due to the limited historical characterization of these viruses (Olsen et al., 2006; Wille et al., 2011; Gambaryan et al., 2012; Gambaryan et al., 2018; Wille et al., 2018). Additionally, a seasonal human IAV was included for comparison [A/Netherlands/213/2003 (H3N2)]. This virus is of the same subtype as one of the studied mallard viruses. The human IAV was cultivated in Madin-Darby canine kidney (MDCK) cells, whereas the studied AIVs were obtained from cloacal swabs, and propagated twice in embryonated chicken eggs. Cultivated viruses were inactivated and FITC-labeled as earlier described (Van Riel et al., 2007).

### Virus Histochemistry

The virus host and tissue attachment were studied using virus histochemistry as earlier described (Van Riel et al., 2007). In brief, tissue slides were deparaffinized in xylene, hydrated in graded alcohols to distilled water, and blocked for endogenous peroxidase in 0.3% hydrogen peroxide. Each slide was incubated overnight at 4°C with 50 HAU of purified formalin fixed FITC-labeled IAV or 1 × PBS (Medicago AB, Uppsala, Sweden) as negative control. FITC-labeled viruses were detected by a peroxidase labeled α-FITC rabbit polyclonal antibody (#ab19492, Abcam, Cambridge, United Kingdom). The signal was amplified by a tyramide signal amplification kit (PerkinElmer AB, Upplands Väsby, Sweden). Peroxidase signal was revealed with 3-amino-9-ethyl-carbazole (Sigma-Aldrich AB, Stockholm, Sweden). Tissues were counterstained with hematoxylin (Sigma-Aldrich), mounted with Vision Mount (Thermo Scientific) and scanned using Aperio Scanscope AT2 (Aperio Technologies, CA, United States). Two independent observers individually scored all digital images. The score given was based on the relative number of virus stained cells for each cell type. The percentage of stained cells of a given cell type in each tissue was scored according to a 6-tiered scale: 0 – <1% stained cells, 1 – 1–10% stained cells, 2 – 11–25% stained cells, 3 – 26–50% stained cells, 4 – 51–75% stained cells, and 5 – >75% stained cells.

### Lectin Staining

Consecutive TMA sections were stained with either 4 μg/mL *Maackia amurensis* lectin II (MAA-II) (BioNordika AB, Stockholm, Sweden) with tropism for α2,3-linked SA or 2 μg/mL *Sambucus nigra* lectin (SNA) (BioNordika) with tropism for α2,6-linked SA from Vector Laboratories. Bound lectins were detected using the Vectastain ABC-AP kit (BioNordika) together with the ImmPACT Vector Red Alkaline Phosphatase Substrate Kit (BioNordika). Lectin stained tissue specimens were counterstained, mounted, scanned, and scored as described above.

### Heatmaps

Heatmaps of obtained staining scores were constructed using the pheatmap R package (Neuwirth, 2014; Wickham, 2014, 2018; R Core Team, 2016; RStudio Team, 2016; Kolde, 2018). Both dendrograms were constructed using Canberra distance measure and UPGMA complete clustering. For overview and readability purpose the heatmaps were based on the maximum score obtained for each species per tissue, since the primary goal of the present study was to qualitatively assess whether any virus attachment could be observed to the studied tissues and avian species. Detailed staining scores are presented in Supplementary Table S1.

## Results

### Virus Staining, Avian Viruses

The PVA was studied in 26 different bird species of seven different orders by virus histochemistry with four LPAIVs of different origins: mallard H3N2, mallard H6N1, ruddy turnstone H12N5, and black-headed gull H16N3 and one human seasonal IAV, H3N2. The PVA was consistent between individuals of the same species, but varied between bird species, as well as between trachea and colon tissues. Most widespread attachment was observed in the tracheae. Detailed individual scores per cell type per species per tissue are displayed in the Supplementary Table S1.

From a host phylogeny point of view, the avian order with the over-all most abundant virus attachment to trachea was Anseriformes (>75% AIV positive stained cells as determined from the four AIVs studied, including both ciliated and goblet cells). The order with the least AIV attachment to trachea was Columbiformes (<10% AIV positive stained cells, including both ciliated and goblet cells). Generally, much less attachment was observed to colon than to trachea. Mallards differed from the other duck species by showing extensive AIV attachment to colon.

**FIGURE 1:**
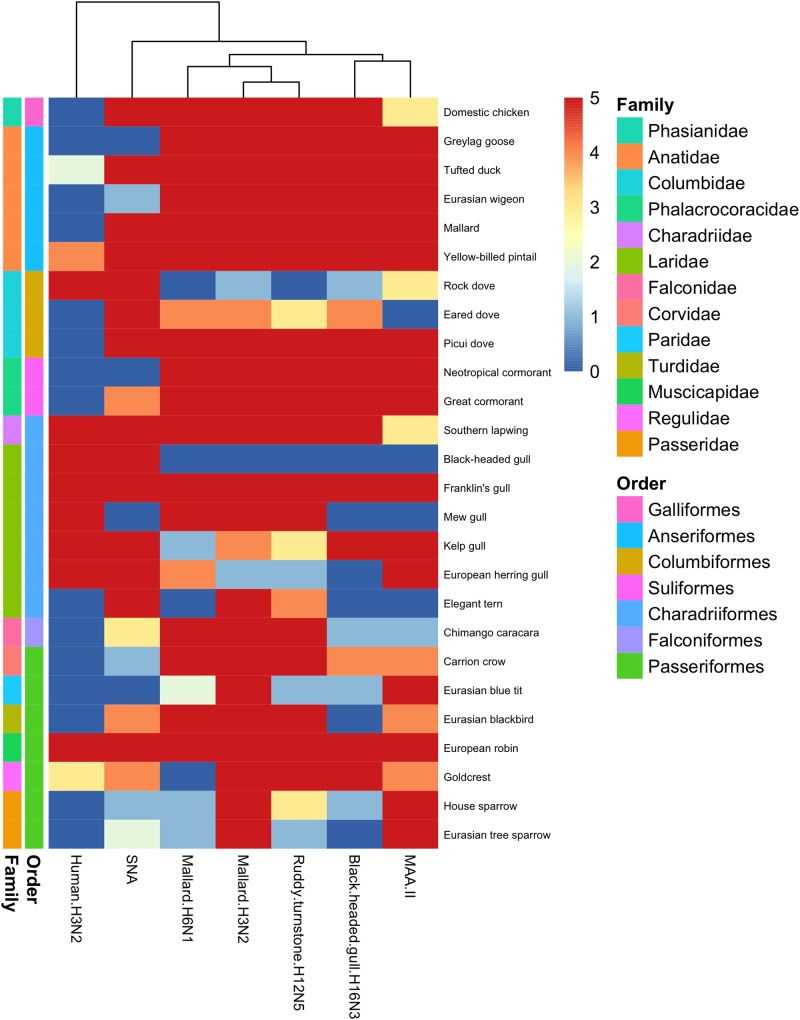
Heatmap of histochemistry staining of avian tracheae TMAs. The heatmap was constructed based on the maximum score obtained for each species.

**FIGURE 2:**
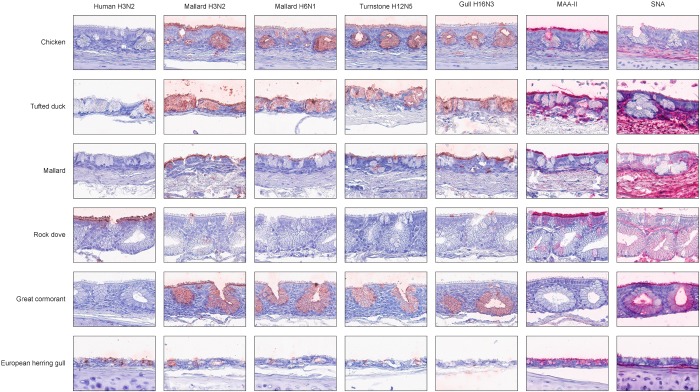
Representative histochemistry images of stained tracheae TMAs. Red color indicates virus/lectin staining. The cells were counterstained with hematoxylin (blue).

**FIGURE 3:**
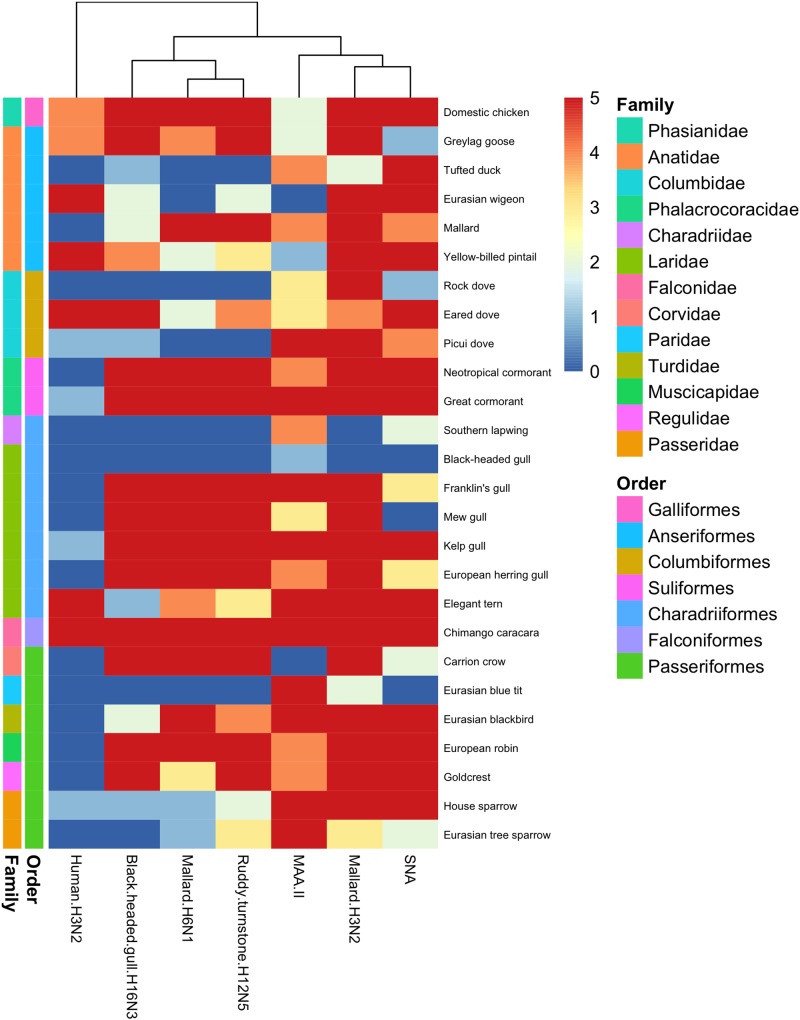
Heatmap of histochemistry staining of avian cola TMAs. The heatmap was constructed based on the maximum score obtained for each species.

**FIGURE 4:**
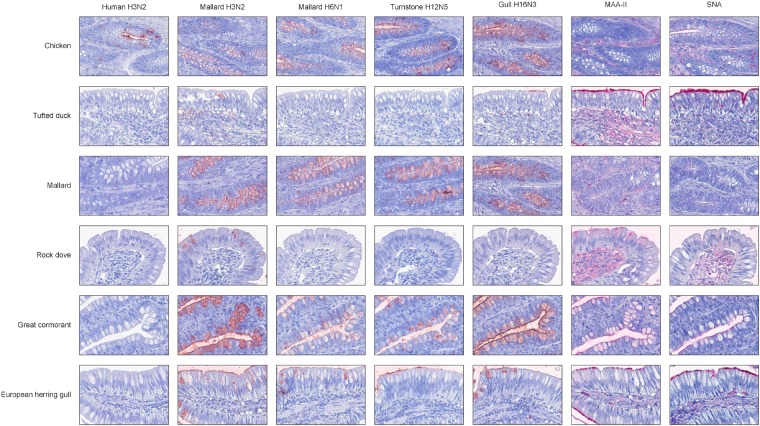
Representative histochemistry images of stained cola TMAs. Red color indicates virus/lectin staining. The cells were counterstained with hematoxylin (blue).

In general, there was a high similarity in the PVA between the avian viruses. In particular, they were very coherent in their PVA to trachea, although the black-headed gull H16N3 virus showed more restricted attachment in contrast to the other avian viruses. There was extensive attachment to chicken, Anseriformes, and cormorants, but somewhat variable to Charadriiformes, as shown in the overview heatmap of trachea staining (Figure 1). No or very limited attachment was observed to rock dove and the two larger gull species (kelp gull and European herring gull). The two mallard viruses and the ruddy turnstone H12N5 virus showed abundant attachment to the tracheae of the two cormorant species (Suliformes), several of the charadriiform species, and to chicken. In these species, the viruses attached both to ciliated epithelial cells and to goblet cells. Representative images of stained trachea samples are displayed in Figure 2. A detailed scoring table is presented in the Supplementary Table S1. The attachment patterns in colon were more variable (Figure 3). All avian viruses attached abundantly to all cell types in the cola of chicken, Franklin’s gull, and mew gull and, to a lesser extent, to goblet and/or crypt cells of greylag goose, the two cormorant species, kelp gull, herring gull, and elegant tern (Figure 3). The two mallard viruses (H3N2 and H6N1) and the ruddy turnstone H12N5 virus attached strongly to all cell types in the cola of mallard and chimango caracara. The mallard H3N2 virus had the broadest PVA of the different investigated AIVs to both trachea and colon. The mallard H6N1 and the ruddy turnstone H12N5 virus had very similar average PVA scores, but somewhat lower attachment signal than the mallard H3N2 virus. Representative images of stained colon samples are displayed in Figure 4.

Among the passerine species, the mallard H3N2 virus showed extensive attachment to trachea from all investigated species (Figure 1). European robin (*Erithacus rubecula*) was the single investigated species with the most extensive virus attachment observed to trachea. The least AIV attachment to passerine trachea was observed from black-headed gull H16N3 virus and the single investigated passerine species with the least virus attachment observed was Eurasian tree sparrow (*Passer montanus*). In passerine colon the mallard H3N2 virus showed the most abundant attachment (maximum attachment to all investigated passerine species except Eurasian blue tit (*Cyanistes caeruleus*) and Eurasian tree sparrow), whereas the black-headed gull H16N3 virus showed the least abundant attachment (Figure 3). Among the investigated cola from passerines, the most abundant virus attachment was observed to carrion crow (*Corvus corone*) and European robin. The investigated passerine species with the least virus attachment observed to colon was Eurasian blue tit.

### Virus Staining, Human Virus

The attachment pattern of the human H3N2 virus differed clearly from that of the avian viruses (Figure 1, 3). This virus showed intense tracheal attachment to all Charadriiformes species examined except elegant tern. It also attached abundantly to trachea of yellow-billed pintail and rock dove. Apart from this, it showed very limited tracheal attachment to most investigated species. Some attachment was observed in the cola of chicken as well as in some wild bird species, including chimango caracara, greylag goose, Eurasian wigeon, yellow-billed pintail, and eared dove, especially in crypt cells. Generally, very limited virus attachment was observed with the human H3N2 virus to tracheae from passerine species, and European robin and goldcrest (*Regulus regulus*) were the only species with any virus attachment observed (Figure 1). The human H3N2 virus had even less attachment to cola from passerine species with only very limited attachment observed to house sparrow (*Passer domesticus*) (Figure 3).

### Lectin Staining

Lectin histochemistry was performed on all avian tissues with the lectins MAA-II and SNA. In trachea, there was clear correlation between MAA-II binding and the PVA of the studied AIVs, as illustrated by the dendrogram in Figure 1. The SNA binding pattern was instead intermediate to the PVA of the human H3N2 virus and the studied AIVs. This pattern was less clear in staining of colon tissues as can be seen in Figure 3. As a matter of fact, the attachment patterns of the two lectins clustered in the colon dendrogram in the avian part of the dendrogram, whereas the human H3N2 virus diverged due to a reduced degree of colon attachment compared to the investigated AIVs and lectins.

## Discussion

Field surveillance studies of various avian orders ranging from Anseriformes to Passeriformes have revealed AIV tropism toward aquatic bird orders, with the highest prevalence reported in Anseriformes (Munster et al., 2007). Additionally, despite the high number of possible HA and NA combinations, a few subtypes dominate in wild ducks and certain HA subtypes are associated with particular host taxa, e.g., H13 and H16 mainly associated with Laridae (Krauss et al., 2004; Olsen et al., 2006; Munster et al., 2007; Wallensten et al., 2007; Huang et al., 2014; Arnal et al., 2015; Wille et al., 2018). Based on the reported surveillance data, it was hypothesized that the studied AIVs would predominantly attach to tissues from bird species of the same order as the host from which they were isolated (Munster et al., 2007). In the present study, this turned out to not be the case. Extensive virus attachment to trachea by all studied AIVs was observed in all investigated avian orders (Figure 1). Unexpectedly, the black-headed gull H16N3 virus did attach to neither the black-headed gull trachea nor colon tissue. However, tissues from only a single individual of black-headed gull was available for this study and it is possible that more virus attachment would had been observed if a larger tissue section had been stained from this individual or a larger set of black-headed gull tissue donors had been used. On the other hand, this virus attached abundantly to all investigated cell types of Franklin’s gull (tissues from four individuals studied), which is a close relative of the black-headed gull (BirdLife International, 2018). Additionally, the gull H16N3 virus attached abundantly to colon of the other studied gull species. IAVs of H16 subtype are mainly found in gulls (Munster et al., 2007; Wille et al., 2011; Höfle et al., 2012), however, the gull H16N3 virus attached abundantly to several anseriform birds (both to trachea and colon) in addition to the gulls. Similar to the black-headed gull H16N3 virus, the ruddy turnstone H12N5 virus was isolated from a charadriiform host. Although tissues from ruddy turnstone were not included in this study, the H12N5 virus, surprisingly, showed less attachment to charadriiform than anseriform tissues (especially to trachea). Yet, H12 AIVs are reported to be rare in anseriform hosts (Krauss et al., 2004; Munster et al., 2007; Wille et al., 2011, 2018). Hence, based on the attachment patterns observed in the current study, the host selectivity of the Charadriiformes specific AIVs (i.e., subtype H16) and other subtypes rare in anseriform hosts (e.g., subtype H12) does not appear to be attributed to tissue attachment, but rather to other factors of the infection process.

There was coherence between the attachment patterns of the different AIVs in the current study, with often more than one virus attaching to the same tissue to a similar degree (see Figure 1, 3 and Supplementary Table S1). In colon, the observed attachment patterns varied in terms of the number of cells involved from none, via intermediate to high, whereas in trachea the observed attachment was more discrete (mainly attachment to no or very many cells). Thus, it seems as there are larger variations in the AIVs’ attachment ability to colon as compared to trachea. Whether this is an effect of variations in the structure of the glycans expressed at the cell surfaces in the different tissues, or if it is due to other factors, needs further investigation. The single avian species with the most AIV attachment to both trachea and colon was the domestic chicken, which had maximum staining score in all studied cell types for all studied AIVs except in tracheal ciliated epithelial cells for the black-headed gull H16N3 virus. This finding is well in line with reports of several AIV subtypes circulating in chickens (Senne et al., 2003; Peng et al., 2013).

Extensive attachment of human seasonal H3N2 virus was observed to trachea of eight species (1 columbiform, 6 charadriiform, and 1 passeriform species). Accordingly, these birds also showed abundant SNA staining, indicating display of α2,6-linked SA. However, extensive SNA staining was not fully predictive of human H3N2 virus attachment, since the tracheae of additional anseriform and columbiform species were extensively stained by SNA, but were negative for human H3N2 virus attachment (Figure 1, 3). This is consistent with previous observations of discrepancies between IAVs attachment and lectin staining (Ellström et al., 2009; Jourdain et al., 2011; Costa et al., 2012; de Graaf and Fouchier, 2014). In colon, staining by the human H3N2 virus was observed in the crypt cells of eight species including chicken.

Although the tissue panel constituted avian species from both the New and Old Worlds, there was no staining trend based on the geographical origin of the sampled species. In particular, the tissue panel comprised four rock doves sampled in Chile and five sampled in Sweden. Despite the geographical distance between these two subpopulations of rock dove, they generally stained very cohesively. Moreover, the virus panel comprised avian viruses isolated both in the Nearctic and Western Palearctic (see section “Materials and Methods,” “Virus Panel”). Noteworthy, the Old World viruses attached intensively to several Neotropical species and the New World virus attached intensively to several Western Palearctic species. As a general conclusion, the phylogeny of the avian species was a better predictor of virus attachment than the geographical origin of both the birds and the viruses. These findings suggest that observed phylogenetic differences in Old and New World AIVs are rather due to limited genetic exchange, than to the tissue attachment of the viruses.

In birds of the orders Columbiformes and Falconiformes, lectin stainings indicated the presence of SA structures in epithelial tissues. Additionally, virus histochemistry revealed virus attachment to the same tissues of these species. Yet, these orders are not usually regarded as important in the ecology of AIVs (Olsen et al., 2006). Historical records of AIV isolates are heavily biased toward sampling efforts made in aquatic environments and it has been suggested that the focus of AIV surveillance should be expanded beyond Anseriformes and Charadriiformes (Krauss et al., 2004; Wallensten et al., 2007; Caron et al., 2017). Passeriformes is the largest avian order, constituting more than half of all extant avian species (BirdLife International, 2018). In the current study, seven members of Passeriformes were investigated for their virus attachment. As a terrestrial order, Passeriformes are not generally regarded as important for the ecology of AIVs among wild birds (Olsen et al., 2006). However, in the current study carrion crow and European robin showed extensive attachment patterns (both in trachea and colon) with the studied viruses. Both carrion crow and European robin are ground dwelling birds that are likely to come in contact with e.g., free-range domestic poultry, suggesting that these species have the potential to serve as bridge species between e.g., anseriform or charadriiform species and domestic poultry. On the other hand, sampling efforts made on terrestrial orders e.g., Passeriformes, have revealed very low prevalence of AIVs, but positive individuals have been detected in e.g., Acrocephalidae, Locustellidae, Hirundinidae, Sylviidae, Muscicapidae, Passeridae, Motacillidae, and Emberizidae (Munster et al., 2007; Gronesova et al., 2008; Peterson et al., 2008; Slusher et al., 2014). Additionally, several of these families include long distance migratory bird species that could potentially carry AIVs over long geographical distances, if infected during migration. Experimental studies have reported large deviations in clinical outcome between different passerine bird species experimentally infected by highly pathogenic AIVs, ranging from very limited symptoms to 100% mortality (Perkins and Swayne, 2003; Breithaupt et al., 2011). Thus, terrestrial avian orders (e.g., Passeriformes) are not resistant to AIV infection, but the low prevalence of AIVs reported in passerines might rather be due to lack of exposure, as AIVs are more commonly associated with aquatic environments. Indeed, AIV has been reported to be persistent in water for months (Stallknecht et al., 2010). Many avian species living in aquatic environments (e.g., mallards) have developed a tolerance toward AIV infection, showing limited or no clinical signs of infection, but when AIVs are introduced in naïve species, the clinical outcome might be severe (Pantin-Jackwood and Swayne, 2009). Yet, the evolutionary distance between birds occupying a mutual habitat might be large. In the case of dabbling ducks (e.g., mallards) vs. diving ducks (e.g., tufted ducks) the divergence point was for approximately 5 million years ago and for Anseriformes (e.g., ducks and geese) vs. Charadriiformes (e.g., gulls and shorebirds) the divergence time was approximately 72 million years ago (Prum et al., 2015). Such long time frames may have created biological barriers due to host differentiation for IAVs interfering with the transmission among different avian taxa such as for IAVs of the subtypes H13 and H16 (Krauss et al., 2004; Olsen et al., 2006; Munster et al., 2007). Indeed, the host restriction of AIV subtypes H13 and H16 has been suggested to be due to specific properties of the H13 and H16 internal proteins (Tonnessen et al., 2013). Still, IAVs of the subtypes H1, H3, H4, and H6 are often isolated from both anseriform and charadriiform hosts (Krauss et al., 2004; Olsen et al., 2006; Munster et al., 2007; Wallensten et al., 2007).

It should be highlighted that virus histochemistry only provides information on host cell attachment, but not ability to replicate. Host cell attachment is an obligate criterion in the virus’ replication cycle, but successful virus replication is dependent on several consecutive steps downstream of host cell attachment. Additional factors apart from the hemagglutinin subtype, virus isolation host species and the conformation of SA contribute to the restrictions and dynamics of IAVs circulation. On the other hand, *in vitro* IAV infection experiments with cell lines have illustrated the importance of the cell type, cell lines vs. primary cells and the differentiation state of the cells used (Chan et al., 2013; de Graaf and Fouchier, 2014). Moreover, careful precautions should be taken to avoid passaging virus isolates through excessive propagation/purification systems, as this risks the selection of non-wild type phenotypes (Gambaryan et al., 2005; Järhult et al., 2015). Further investigations are needed to better correlate IAV attachment and *in vitro* replication studies.

## Conclusion

In conclusion, historically Anseriformes (e.g., mallards) and Charadriiformes (e.g., gulls) have been regarded as the main reservoir of AIVs (Olsen et al., 2006). However, in recent years the community concept of AIVs has been postulated (Caron et al., 2017). In surveillance studies, there might exist a sampling bias since sampling efforts have been directed toward cloacal swabbing and not oropharyngeal sample collection (Munster et al., 2007; Wallensten et al., 2007). Method evaluation studies have reported varying ratios of AIV positivity in oropharyngeal vs. cloacal samples, and this observation seems to be host species dependent (Ellström et al., 2008; Munster et al., 2009; Hoye et al., 2010). Similarly, in the present study AIV attachment was observed to several different avian species and orders independent of the avian species of virus isolation. AIV is mainly associated with colon in mallards (Webster et al., 1978; Olsen et al., 2006; Munster et al., 2007; Wallensten et al., 2007; Ellström et al., 2008; Costa et al., 2012; Bröjer et al., 2013; Lindskog et al., 2013). However, in the current study extensive tracheal attachment was observed in several non-mallard species, indicating the importance of the respiratory tract when investigating non-mallard species.

## Ethics Statement

Bird tissue sampling procedures were approved by the Swedish Environmental Protection Agency (permits numbers 412-6267- 08NV/412-5977-08NV), the Swedish Board of Agriculture (permit numbers 74-08/43-09), the Chilean Agriculture Ministry (permit number 1-25-2008), and the Ethics Committee of the Veterinary University of Concepción (permit number CE1-2006). All experimental procedures were performed in accordance with relevant guidelines and regulations.

## Author Contributions

PEr, CL, JW, DG-A, JJ, ÅL, BO, EJ, and PEl designed the study. PEl prepared the labeled viruses. JW, DG-A, EJ, and PEl made the tissue collections. PEr, CL, VL-L, and EJ contributed to the data collection and analysis. PEr, CL, JW, JJ, EJ, and PEl prepared the manuscript and all co-authors approved the final manuscript.

## Conflict of Interest Statement

The authors declare that the research was conducted in the absence of any commercial or financial relationships that could be construed as a potential conflict of interest.

## Supplementary Material

TABLE S1 Individual staining scores 0 – <1% stained cells, 1 – 1–10% stained cells, 2 – 11–25% stained cells, 3 – 26–50% stained cells, 4 – 51–75% stained cells, and 5 – >75% stained cells. N/A, not applicable. n, number of stained individuals.
