# Supplementary material for: Attachment Patterns of Human and Avian Influenza Viruses to Trachea and Colon of 26 Bird Species – Support for the Community Concept
Source: Front Microbiol. 2019 Apr 18;10:815. doi: 10.3389/fmicb.2019.00815 (PMC6482220; doi:10.3389/fmicb.2019.00815)
Supplement: TABLE S1 — Individual staining scores 0 – <1% stained cells, 1 – 1–10% stained cells, 2 – 11–25% stained cells, 3 – 26–50% stained cells, 4 – 51–75% stained cells, and 5 – >75% stained cells. N/A, not applicable. n, number of stained individuals. [file Table_1.pdf]

		Trachea cell type			Colon cell type			
**Reagent**	**Avian species**	**ciliated**	**goblet**	**n**	**epithelial**	**goblet**	**crypt**	**n**
**Human H3N2**	Domestic chicken	N/A;0;0;0;0	N/A;0;0;0;0	4	0;0;0;N/A;N/A	0;0;0;N/A;N/A	2;1;0;4;N/A	4
	Greylag goose	0;0;0;N/A	0;0;0;N/A	3	0;0;0;N/A	0;0;0;N/A	4;4;3;N/A	3
	Tufted duck	0;0;0;0	2;0;0;0	4	0;0;0;0	0;0;0;0	0;0;0;0	4
	Eurasian wigeon	0;0;0;0	0;0;0;0	4	0;0;0;0	0;1;2;0	0;5;5;1	4
	Mallard	0;0;0;N/A	0;0;0;N/A	3	0;0;0;0;N/A	0;0;0;0;N/A	0;0;0;0;N/A	4
	Yellow-billed pintail	4;N/A;0;N/A	3;N/A;0;1	3	N/A;0;0;5	N/A;1;1;5	0;3;5;5	4
	Rock dove	5;5;5;5;5;5;5;5;5	1;1;1;1;1;2;2;2;2	9	0;0;0;0;0;0;0;0;N/A	0;0;0;0;0;0;0;0;N/A	0;0;0;0;0;0;0;0;N/A	8
	Eared dove	0;0;0;0	0;0;0;0	4	2;1;1;1	1;2;0;1	5;N/A;3;3	4
	Picui dove	0;0;0;0	0;0;0;0	4	0;N/A;0;0	0;N/A;0;1	0;N/A;0;1	3
	Neotropical cormorant	0;N/A;0;0	0;N/A;0;0	3	0;0;0;0	0;0;0;0	0;0;0;0	4
	Great cormorant	0;0;0;0;0;0	0;0;0;0;N/A;0	6	0;0;0;0;0;0	1;1;1;1;0;0	1;1;1;0;1;1	6
	Southern lapwing	N/A;5;5;5	N/A;5;5;5	3	0;0;0;0	0;0;0;0	0;0;0;0	4
	Black-headed gull	5	5	1	0	0	0	1
	Franklin's gull	5;5;5;0	5;5;5;N/A	4	0;0;0;0	0;0;0;0	0;0;0;0	4
	Mew gull	0	5	1	N/A	N/A	0	1
	Kelp gull	N/A;5;5;N/A	N/A;5;5;N/A	4	0;0;0;0	0;0;0;0	0;0;0;1	4
	European herring gull	0;0;0;0;0	N/A;5;5;5;5	5	0;0;0;0;0	0;0;0;0;0	0;0;0;0;0	5
	Elegant tern	0	0	1	5	1	5	1
	Chimango caracara	0;0;0;0	0;0;0;0	4	1;3;5;2	0;1;5;0	3;3;5;3	4
	Carrion crow	0;0;0;0	0;0;0;0	4	0;0;0;0	0;0;0;0	0;0;0;0	4
	Eurasian blue tit	0;0;0;0	0;0;0;0	4	0;0;0;0	0;0;0;0	0;0;0;0	4
	Eurasian blackbird	0;0	0;0	2	0;0	0;0	0;0	2
	European robin	1;3;1;1	4;3;5;4	4	0;0;0;0	0;0;0;0	0;0;0;0	4
	Goldcrest	0;0;0;0	0;3;0;0	4	0;0;0;0	0;0;0;0	0;0;0;0	4
	House sparrow	0;0;0;0;0	0;0;0;0;0	5	1;1;1;0;0	1;1;1;0;0	0;0;0;N/A;0	5

	Eurasian tree sparrow	0;0;0	0;0;0	3	0;0;0	0;0;0	0;0;0	3
**Mallard H3N2**	Domestic chicken	N/A;5;N/A;5;5	N/A;5;N/A;5;5	3	5;5;5;N/A;N/A	5;5;5;N/A;N/A	5;5;5;5;N/A	4
	Greylag goose	5;5;5;5	5;5;5;5	4	4;1;5;N/A	5;3;5;N/A	2;1;5;N/A	3
	Tufted duck	5;5;5;5	5;5;5;5	4	2;0;0;0	1;1;0;0	1;0;0;0	4
	Eurasian wigeon	5;5;5;5	5;5;5;5	4	0;N/A;0;0	5;5;4;0	0;0;0;0	4
	Mallard	5;5;5;5	5;5;5;5	4	5;5;5;5;N/A	5;5;5;5;N/A	5;5;5;5;N/A	4
	Yellow-billed pintail	5;N/A;5;5	5;N/A;5;5	3	N/A;0;4;0	N/A;4;5;3	1;0;2;1	4
	Rock dove	0;0;0;0;0;0;0;0;0	0;0;0;0;0;0;0;0;1	9	0;0;0;0;0;0;0;0;N/A	0;4;0;2;4;3;5;4;N/A	0;0;0;0;0;0;0;0;N/A	8
	Eared dove	3;4;2;4	4;4;3;3	4	1;0;0;0	3;1;1;1	4;N/A;1;0	4
	Picui dove	5;5;5;5	2;1;0;1	4	5;N/A;5;5	1;N/A;0;3	0;N/A;1;1	3
	Neotropical cormorant	4;5;4;5	5;5;5;5	4	2;4;0;4	5;5;0;5	4;2;0;4	4
	Great cormorant	5;5;5;5;5;5	5;4;5;5;N/A;5	6	5;5;0;0;0;0	3;3;5;2;0;5	4;4;5;0;0;4	6
	Southern lapwing	5;5;5;N/A	1;4;4;N/A	3	0;0;0;0	0;0;0;0	0;0;0;0	4
	Black-headed gull	0	0	1	0	0	0	1
	Franklin's gull	5;5;5;5	2;1;5;5	4	0;5;5;5	0;5;5;5	0;5;5;5	4
	Mew gull	5	5	1	5	5	5	1
	Kelp gull	N/A;0;0;0	N/A;1;0;4	3	0;2;4;0	1;0;4;1	0;5;5;2	4
	European herring gull	N/A;0;1;N/A;0	N/A;0;1;N/A;0	3	0;3;0;0;5	3;4;0;1;5	3;5;1;5;4	5
	Elegant tern	5	0	1	1	5	1	1
	Chimango caracara	5;5;5;N/A	5;5;N/A;N/A	3	5;5;5;5	5;4;5;5	4;5;5;5	4
	Carrion crow	5;5;5;5	5;5;3;3	4	5;5;5;5	5;5;5;5	5;5;5;5	4
	Eurasian blue tit	4;3;2;5	4;3;3;3	4	0;0;0;1	0;0;2;1	0;0;0;0	4
	Eurasian blackbird	5;5	5;5	2	5;5	5;5	5;5	2
	European robin	5;5;5;5	5;5;5;5	4	5;5;5;5	5;5;5;5	4;5;5;5	4
	Goldcrest	4;5;2;4	2;4;1;4	4	5;2;1;4	0;0;0;0	0;0;0;0	4
	House sparrow	5;4;4;1;1	5;5;2;2;1	5	5;5;5;3;5	0;4;3;0;3	1;4;3;N/A;3	5
	Eurasian tree sparrow	3;5;2	0;2;0	3	3;2;1	3;1;0	2;1;0	3

**Mallard H6N1**	Domestic chicken	N/A;5;N/A;5;5	N/A;5;N/A;5;5	3	5;5;5;N/A;N/A	5;5;5;N/A;N/A	5;5;5;5;N/A	4
	Greylag goose	5;5;5;5	5;5;5;5	4	1;0;1;N/A	4;0;3;N/A	0;0;3;N/A	3
	Tufted duck	5;5;5;5	5;5;4;5	4	0;0;0;0	0;0;0;0	0;0;0;0	4
	Eurasian wigeon	5;5;5;5	5;5;5;5	4	0;0;0;0	0;0;0;0	0;0;0;0	4
	Mallard	N/A;5;N/A;5	N/A;5;N/A;5	2	5;5;5;5;N/A	5;5;5;5;N/A	5;5;5;5;N/A	4
	Yellow-billed pintail	5;N/A;5;5	5;N/A;5;5	3	N/A;0;0;0	N/A;1;2;1	0;0;0;0	4
	Rock dove	0;0;0;0;0;0;0;0;0	0;0;0;0;0;0;0;0;0	9	0;0;0;0;0;0;0;0;N/A	0;0;0;0;0;0;0;0;N/A	0;0;0;0;0;0;0;N/A	8
	Eared dove	3;2;2;2	3;0;2;4	4	0;0;0;0	0;0;0;0	2;N/A;0;0	4
	Picui dove	4;5;4;4	1;1;0;0	4	0;N/A;0;0	0;N/A;0;0	0;N/A;0;0	3
	Neotropical cormorant	5;5;4;5	5;5;5;5	4	N/A;0;0;5	5;4;0;5	3;1;0;5	4
	Great cormorant	5;5;5;5;5;5	5;5;5;5;5;5	6	5;5;1;0;0;0	4;4;5;2;0;5	5;4;3;1;0;5	6
	Southern lapwing	5;5;0;5	3;5;2;N/A	4	0;0;0;0	0;0;0;0	0;0;0;0	4
	Black-headed gull	0	0	1	0	0	0	1
	Franklin's gull	5;5;5;5	3;5;5;0	4	0;5;5;5	0;5;5;5	0;5;5;5	4
	Mew gull	5	4	1	5	5	5	1
	Kelp gull	N/A;0;0;0	N/A;1;0;0	3	0;1;3;0	0;0;4;0	0;5;5;1	4
	European herring gull	2;0;0;0;0	N/A;0;0;4;2	5	0;1;0;0;4	0;4;0;0;2	1;5;1;5;5	5
	Elegant tern	0	0	1	0	4	0	1
	Chimango caracara	5;5;5;5	5;5;5;5	4	5;5;5;5	5;5;5;5	5;5;5;5	4
	Carrion crow	1;2;1;1	5;4;1;1	4	5;5;1;2	5;3;3;3	5;3;1;3	4
	Eurasian blue tit	2;0;0;0	2;0;0;1	4	0;0;0;0	0;0;0;0	0;0;0;0	4
	Eurasian blackbird	3;5	5;5	2	3;5	3;5	1;0	2
	European robin	4;5;5;5	4;5;5;5	4	2;3;4;4	4;5;4;4	0;2;3;1	4
	Goldcrest	0;0;0;0	0;0;0;0	4	3;1;0;1	0;0;0;0	0;0;0;0	4
	House sparrow	0;0;0;0;0	0;1;0;0;0	5	1;0;0;0;0	0;0;1;0;0	0;1;1;N/A;0	5
	Eurasian tree sparrow	0;0;0	0;1;0	3	1;1;0	0;0;0	0;0;0	3
**Ruddy turnstone**	Domestic chicken	N/A;5;N/A;5;N/A	N/A;5;N/A;5;N/A	2	5;5;5;N/A;N/A	5;5;5;N/A;N/A	5;5;5;5;N/A	4

**H12N5**								
	Greylag goose	5;N/A;5;5	5;N/A;5;5	3	0;0;5;N/A	5;1;5;N/A	3;5;5;N/A	3
	Tufted duck	5;5;5;5	5;5;N/A;5	4	0;0;0;0	0;0;0;0	0;0;0;0	4
	Eurasian wigeon	5;5;5;5	5;5;5;5	4	0;0;0;0	0;1;2;0	0;0;0;0	4
	Mallard	N/A;5;N/A;5	N/A;5;N/A;3	2	5;5;5;5;N/A	5;5;5;5;N/A	4;4;4;4;N/A	4
	Yellow-billed pintail	5;N/A;5;5	5;N/A;5;4	3	N/A;0;1;0	N/A;2;3;2	0;0;0;1	4
	Rock dove	0;0;0;0;0;0;0;0;0	0;0;0;0;0;0;0;0;0	9	0;0;0;0;0;0;0;0;N/A	0;0;0;0;0;0;0;0;N/A	0;0;0;0;0;0;0;0;N/A	8
	Eared dove	2;2;0;0	3;0;1;1	4	1;0;0;0	0;0;0;0	4;N/A;2;1	4
	Picui dove	5;5;5;5	1;1;1;1	4	0;N/A;0;0	0;N/A;0;0	0;N/A;0;0	3
	Neotropical cormorant	3;3;5;3	5;5;5;5	4	0;0;0;0	5;2;0;5	4;0;0;2	4
	Great cormorant	4;5;5;5;5;5	2;0;5;5;5;5	6	5;5;0;0;0;0	3;3;5;3;1;5	5;3;4;1;0;5	6
	Southern lapwing	4;3;2;N/A	4;4;5;N/A	3	0;0;0;0	0;0;0;0	0;0;0;0	4
	Black-headed gull	0	0	1	0	0	0	1
	Franklin's gull	5;5;5;5	1;4;5;N/A	4	0;5;5;5	0;5;4;5	0;5;5;5	4
	Mew gull	5	5	1	5	5	5	1
	Kelp gull	N/A;0;0;0	N/A;1;0;3	3	0;1;2;0	0;0;3;0	0;5;5;0	4
	European herring gull	N/A;0;0;0;0	N/A;0;1;0;1	4	0;1;0;0;4	0;3;0;0;2	0;5;0;3;5	5
	Elegant tern	4	3	1	0	3	0	1
	Chimango caracara	0;0;0;0	4;2;5;N/A	4	5;5;5;5	5;5;5;1	5;5;5;3	4
	Carrion crow	1;3;2;1	5;3;1;1	4	5;5;3;5	5;3;3;3	4;4;4;4	4
	Eurasian blue tit	1;0;0;0	1;0;0;0	4	0;0;0;0	0;0;0;0	0;0;0;0	4
	Eurasian blackbird	5;5	5;5	2	1;1	4;4	0;0	2
	European robin	5;5;5;5	5;5;5;5	4	1;1;2;3	4;3;5;3	0;1;5;1	4
	Goldcrest	2;3;5;3	0;3;2;5	4	4;1;1;5	0;0;0;0	0;0;1;0	4
	House sparrow	0;0;0;0;1	3;2;1;0;1	5	2;0;1;0;0	0;0;1;0;0	0;2;0;N/A;0	5
	Eurasian tree sparrow	0;1;1	0;1;0	3	3;1;0	0;0;0	0;0;0	3
**Black-headed gull H16N3**	Domestic chicken	N/A;0;N/A;0;0	N/A;5;N/A;5;5	3	5;5;5;N/A;N/A	5;5;5;N/A;N/A	5;5;5;5;N/A	4

	Greylag goose	5;5;5;5	3;N/A;5;5	4	0;1;4;N/A	0;0;1;N/A	5;5;5;N/A	3
	Tufted duck	5;5;5;5	5;5;4;5	4	0;0;0;0	0;0;0;0	0;0;1;0	4
	Eurasian wigeon	N/A;5;5;5	N/A;4;5;5	3	0;0;0;0	0;1;2;0	0;0;0;0	4
	Mallard	5;5;N/A;N/A	5;3;N/A;N/A	2	0;0;0;0;N/A	0;0;0;0;N/A	0;0;2;0;N/A	4
	Yellow-billed pintail	5;N/A;5;5	5;N/A;4;5	3	N/A;0;0;0	N/A;0;0;0	1;2;3;4	4
	Rock dove	0;0;0;0;0;0;0;1;0	0;0;0;0;0;0;0;0;0	9	0;0;0;0;0;0;0;0;N/A	0;0;0;0;0;0;0;0;N/A	0;0;0;0;0;0;0;0;N/A	8
	Eared dove	4;0;0;0	0;0;0;0	4	5;4;5;5	2;2;4;5	5;N/A;5;5	4
	Picui dove	1;5;5;4	0;1;1;3	4	1;N/A;0;0	0;N/A;0;0	0;N/A;0;0	3
	Neotropical cormorant	0;0;0;0	2;2;5;3	4	3;0;0;5	5;0;0;5	5;0;0;5	4
	Great cormorant	0;0;0;0;0;0	5;0;5;4;N/A;5	6	5;5;0;0;0;0	2;2;5;1;1;5	5;4;5;1;0;5	6
	Southern lapwing	5;3;5;5	3;2;4;5	4	0;0;0;0	0;0;0;0	0;0;0;0	4
	Black-headed gull	0	0	1	0	0	0	1
	Franklin's gull	5;5;5;5	5;1;5;N/A	4	0;5;4;5	0;5;5;5	0;5;5;5	4
	Mew gull	0	N/A	1	1	5	5	1
	Kelp gull	N/A;0;0;0	N/A;5;0;2	3	1;5;5;1	0;5;5;1	0;5;5;4	4
	European herring gull	N/A;0;0;0;0	N/A;0;0;0;0	4	1;5;1;0;3	1;5;0;0;3	5;5;5;3;5	5
	Elegant tern	0	0	1	1	1	0	1
	Chimango caracara	0;0;0;0	1;0;0;N/A	4	4;4;5;5	1;1;3;1	4;4;4;5	4
	Carrion crow	0;4;3;1	0;4;3;0	4	0;0;0;1	1;1;0;1	5;3;1;5	4
	Eurasian blue tit	1;0;0;1	1;0;0;0	4	0;0;0;0	0;0;0;0	0;0;0;0	4
	Eurasian blackbird	0;0	0;0	2	1;0	2;1	0;0	2
	European robin	5;5;5;5	5;5;5;5	4	5;5;5;5	5;5;5;5	5;5;5;5	4
	Goldcrest	4;5;N/A;5	4;5;N/A;5	4	5;2;1;4	0;0;0;0	5;5;5;5	4
	House sparrow	0;0;0;N/A;1	1;0;0;N/A;1	5	1;1;0;0;1	0;0;0;0;0	0;0;0;0;0	5
	Eurasian tree sparrow	0;0;0	0;0;0	3	0;0;0	0;0;0	0;0;0	3
**MAA-II**	Domestic chicken	0;1;3;3;1	0;0;0;1;0	5	0;0;1;0;N/A	0;0;0;0;N/A	1;0;0;2;0	5
	Greylag goose	5;N/A;5;N/A	0;N/A;0;N/A	2	1;0;0;N/A	2;0;2;N/A	1;0;0;N/A	3

	Tufted duck	3;N/A;5;5	0;N/A;0;3	3	0;0;0;4	0;0;0;0	0;0;0;0	4
	Eurasian wigeon	N/A;4;N/A;5	N/A;5;N/A;4	2	0;0;0;0	0;0;0;0	0;0;0;0	4
	Mallard	5;5;5;N/A	0;0;0;N/A	3	0;1;0;0;4	0;0;0;2;1	0;0;0;0;1	5
	Yellow-billed pintail	4;N/A;5;N/A	0;N/A;1;0	3	N/A;0;0;0	N/A;0;0;0	1;1;1;1	4
	Rock dove	0;0;0;0;0;0;0;0;0	0;0;3;0;1;0;0;0;0	9	0;1;0;2;0;3;3;1;0	0;0;0;0;0;1;2;1;0	0;0;0;0;0;1;1;0;0	9
	Eared dove	0;0;0;0	0;0;0;0	4	0;0;3;0	0;0;0;0	0;N/A;0;0	4
	Picui dove	0;1;0;0	5;5;5;5	4	5;N/A;5;5	5;N/A;5;4	5;N/A;4;3	3
	Neotropical cormorant	3;4;4;5	2;1;2;2	4	N/A;4;4;2	N/A;0;3;0	4;1;4;0	4
	Great cormorant	5;5;5;5;5;5	5;5;5;5;5;5	6	0;0;4;3;5;3	3;4;4;4;4;3	4;4;5;4;5;4	6
	Southern lapwing	0;0;0;0	2;3;2;1	4	4;3;4;3	3;2;1;1	3;3;2;1	4
	Black-headed gull	0	0	1	1	0	0	1
	Franklin's gull	5;5;5;5	0;0;0;0	4	5;5;5;5	0;0;2;0	4;1;1;1	4
	Mew gull	N/A	N/A	0	3	0	1	1
	Kelp gull	N/A;N/A;5;0	N/A;N/A;0;0	2	4;0;5;1	1;0;0;0	4;1;0;3	4
	European herring gull	N/A;0;0;2;5	N/A;0;0;0;0	4	0;0;0;2;4	0;1;0;0;0	1;1;1;1;4	5
	Elegant tern	0	0	1	5	5	5	1
	Chimango caracara	0;0;1;0	0;0;0;0	4	5;1;3;5	0;0;1;0	0;0;0;1	4
	Carrion crow	2;3;N/A;4	1;3;N/A;4	4	0;0;0;0	0;0;0;0	0;0;0;0	4
	Eurasian blue tit	3;3;4;5	3;3;4;5	4	3;3;1;1	5;5;5;4	5;5;4;3	4
	Eurasian blackbird	N/A;4	N/A;3	2	1;5	1;1	0;1	2
	European robin	5;0;0;1	5;0;0;1	4	3;3;2;3	4;4;3;4	4;4;4;4	4
	Goldcrest	3;2;4;4	2;1;2;3	4	2;4;3;3	2;3;2;2	3;3;1;2	4
	House sparrow	5;5;5;3;N/A	3;3;1;3;N/A	5	5;2;5;5;3	0;2;0;4;5	1;2;0;N/A;1	5
	Eurasian tree sparrow	5;N/A;5	2;N/A;5	3	1;0;0	5;5;0	3;4;0	3
**SNA**	Domestic chicken	5;5;5;5;5	0;3;4;2;0	5	0;1;1;1;N/A	0;0;0;0;N/A	0;1;1;1;5	5
	Greylag goose	0;0;0;N/A	0;0;0;N/A	3	0;0;1;N/A	0;0;0;N/A	0;1;1;N/A	3
	Tufted duck	5;5;5;5	4;4;3;4	4	0;0;1;5	0;0;0;0	0;0;0;0	4
	Eurasian	N/A;N/A;	N/A;N/A;	1	0;0;0;0	0;2;2;0	3;4;5;3	4

	wigeon	N/A;0	N/A;1					
	Mallard	5;5;5;5	N/A;0;0;0	4	0;0;0;0;0	0;0;0;0;0	1;4;4;3;3	5
	Yellow-billed pintail	5;N/A;N/A;5	1;N/A;N/A;0	2	N/A;1;3;4	N/A;1;1;2	3;4;5;5	4
	Rock dove	5;5;5;5;5;5;5;5;5	2;1;2;2;1;3;2;2;2	9	0;0;0;0;0;0;1;0;0	0;0;0;0;0;0;0;0	0;0;0;0;0;0;0;0;0	9
	Eared dove	5;5;5;5	5;5;3;5	4	5;4;5;4	3;4;3;4	5;N/A;4;4	4
	Picui dove	2;5;3;4	1;1;5;5	4	2;N/A;4;3	3;N/A;4;4	4;N/A;3;3	3
	Neotropical cormorant	0;N/A;0;0	0;N/A;0;0	3	N/A;5;4;5	N/A;0;0;0	4;4;4;3	4
	Great cormorant	0;0;4;0;2;1	0;0;0;0;0;0	6	0;0;3;0;5;3	1;1;2;1;1;0	1;1;4;1;3;3	6
	Southern lapwing	5;5;5;5	5;5;5;4	4	1;1;2;1	0;0;2;1	0;0;0;1	4
	Black-headed gull	5	5	1	0	0	0	1
	Franklin's gull	5;5;5;5	5;5;5;5	4	2;3;1;0	3;0;0;0	2;0;1;1	4
	Mew gull	N/A	N/A	0	0	0	0	1
	Kelp gull	5;N/A;5;5	5;N/A;5;5	3	4;0;5;0	0;0;0;0	0;0;0;1	4
	European herring gull	N/A;5;5;5;5	N/A;5;5;5;5	4	0;0;0;2;3	0;0;0;0;0	0;0;0;0;0	5
	Elegant tern	5	4	1	5	3	5	1
	Chimango caracara	0;0;0;0	0;0;3;0	4	5;4;5;5	0;0;1;0	5;5;4;5	4
	Carrion crow	1;1;0;1	0;1;0;0	4	0;0;0;0	1;0;1;2	2;2;1;2	4
	Eurasian blue tit	N/A;0;0;0	N/A;0;0;0	4	0;0;0;0	0;0;0;0	0;0;0;0	4
	Eurasian blackbird	3;2	4;4	2	2;5	0;0	1;3	2
	European robin	5;5;4;3	5;5;4;3	4	0;1;2;4	0;0;0;0	5;4;4;5	4
	Goldcrest	2;2;N/A;N/A	2;4;N/A;N/A	4	0;0;0;5	0;0;0;4	0;0;0;0	4
	House sparrow	0;0;0;0;1	0;0;0;0;1	5	0;0;0;2;0	0;0;0;5;0	0;0;0;N/A;1	5
	Eurasian tree sparrow	1;N/A;0	2;N/A;0	3	2;2;0	0;0;0	0;0;0	3
